# A novel and efficient surgical procedure for pyriform sinus fistulas in children

**DOI:** 10.3389/fped.2024.1297831

**Published:** 2024-05-30

**Authors:** Jiarui Chen, Ying Wang, Chenling Shen, Jing Wang, Xiaoyan Li

**Affiliations:** Department of Otorhinolaryngology-Head & Neck Surgery, Shanghai Children’s Hospital, Shanghai Jiaotong University School of Medicine, Shanghai, China

**Keywords:** children, surgery, pyriform sinus fistulas, pediatric surgery, surgical procedures

## Abstract

**Objective:**

This study aims to evaluate the efficacy of a novel surgical procedure for pyriform sinus fistulas in children via the external cervical approach through the hypopharynx.

**Methods:**

A retrospective analysis was conducted on 71 pediatric patients with pyriform sinus fistula (PSF) who underwent treatment at the Department of Otolaryngology Head and Neck Surgery, Shanghai Children's Hospital, from 2012 July to 2022 July. Surgical treatment of PSF was performed via the external cervical approach through the hypopharynx, with dye instilled through the internal opening to serve as a guide for tract identification.

**Results:**

All the internal orifices were found in the pyriform sinus by direct laryngoscopy in all 71 patients under general anesthesia. Two patients had a postoperative temporary hoarseness that resolved after 3 months. The other patients had no complications such as parapharyngeal abscess and incision infection. All patients were followed up for 23 months–70 months, and no recurrence was found.

**Conclusion:**

The novel surgical procedure of PSF performed via the hypopharynx has advantages such as a short operation path, quick recovery, fewer complications, and a low recurrence rate. This method is a better choice for managing recurrence cases after repeated cauterization endoscopic surgeries, as well as for patients with visible cervical surgical scars or masses.

## Introduction

Pyriform sinus fistula (PSF) is a rare congenital anomaly originating from the remnants of the third or fourth branchial pouch that develops in childhood in approximately 80% of patients (1). Anomalies of the third and fourth branchial clefts account for 3%–10% of all branchial cleft anomalies. PSF commonly presents with symptoms such as recurrent neck swelling, abscess formation, or suppurative thyroiditis. However, the unspecific presentations and rarity of PSF often lead to delayed or misdiagnosed diagnoses. The current main surgical treatment method is endoscopic management by cauterization of the internal orifice; however, this approach carries a high probability of PSF recurrence. The recurrence causes are related to factors such as unresectable fistula or incomplete removal of fistula, failure of mucosal cauterization of the internal orifice to completely seal the fistula, and the continuous existence of fistula and secondary infection (2). In addition, endoscopic surgery may not deal with the scars of abscess incisions and inflammatory masses in the neck. Therefore, it has been proposed that fistulectomy through an external cervical approach is a fundamental measure to prevent recurrent infection (3, 4). Historically, traditional open surgery was routinely performed to excise the entire fistulous tract with/without hemithyroidectomy (5). However, problems such as long path, large trauma, significant postoperative scars, and difficulty in reaching the root of the fistula through the external fistula access result in incomplete fistula removal and postoperative recurrence. Simultaneous hemithyroidectomy can damage the superior thyroid artery and recurrent laryngeal nerve; therefore, it is of great clinical significance to improve the traditional surgical method and find a more effective and minimally invasive surgical one. The authors have improved the previous surgical method, which involves dissecting and completely removing the fistula via the hypopharyngeal route. This study aims to evaluate the efficacy of a novel surgical procedure for pyriform sinus fistulas in children via the external cervical approach through the hypopharynx.

## Materials and methods

### General material

This is a retrospective study of a series of consecutive pediatric patients diagnosed with PSF and treated at the Department of Otolaryngology Head and Neck Surgery, Shanghai Children's Hospital, from 2012 July to 2022 July. We reviewed the medical records including demographics, clinical symptoms, surgical details, and postoperative course of each patient. The inclusion criteria were as follows: (1) clinical diagnosis of PSF, anti-infective treatment or abscess incision and drainage after more than 1 month, and no obvious local symptoms of infection and (2) routine preoperative examination and no contraindications to surgery. Ethical approval was obtained from the Ethics Board of the Shanghai Children's Hospital of Shanghai Jiao Tong University. Written informed consent was obtained from the parents of the patients on behalf of their children.

### Surgical protocol

**Figure 1:**
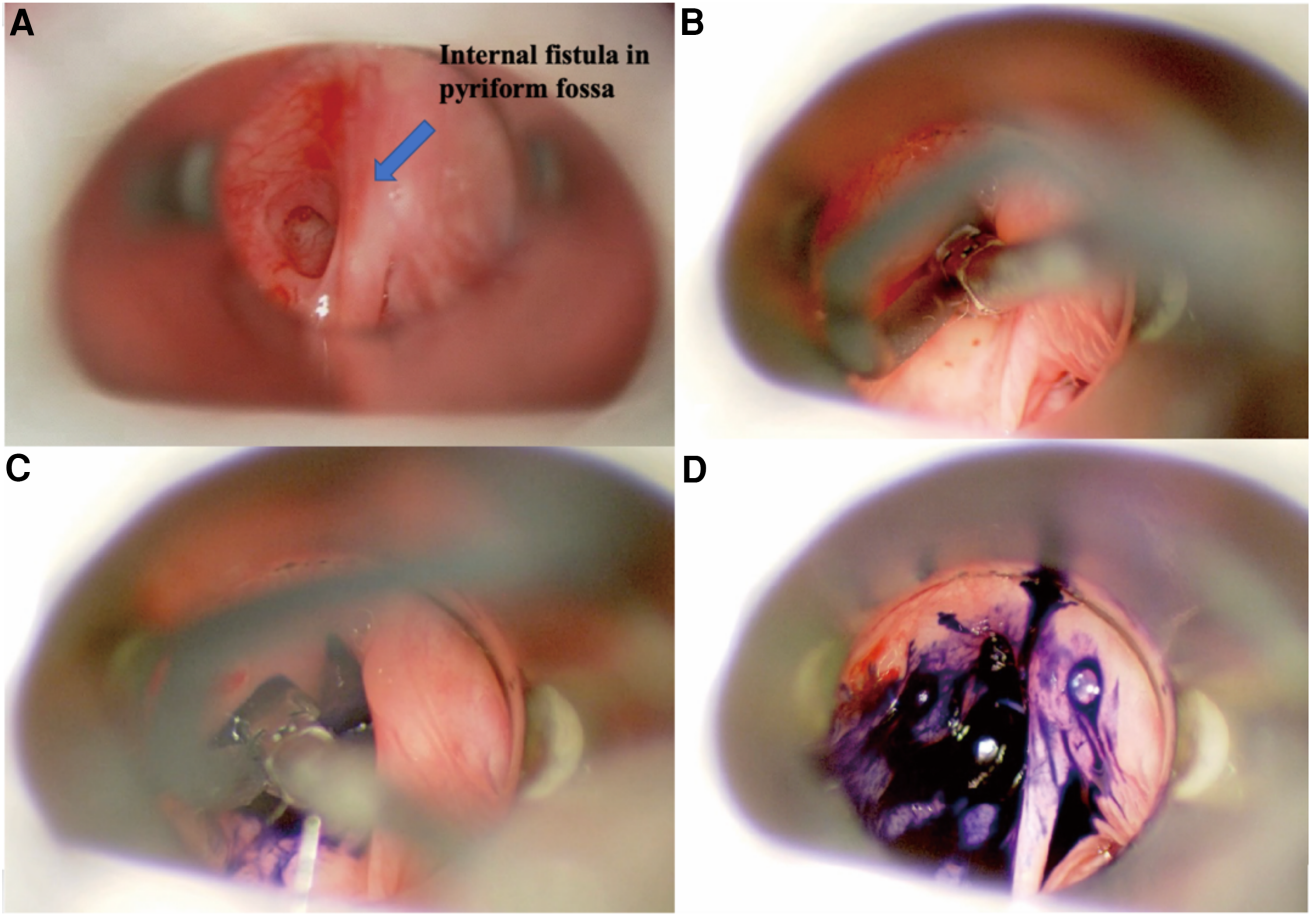
Endoscopic view of left pyriform sinus fistula. (**A**) Direct laryngoscopic exposure of the left internal orifice in the pyriform fossa. (**B**) Blunt-tip gastric tube was inserted into the internal orifice. (**C**) Methylene blue was slowly injected. (**D**) Methylene blue overflowed from the internal orifice.

**Figure 2:**
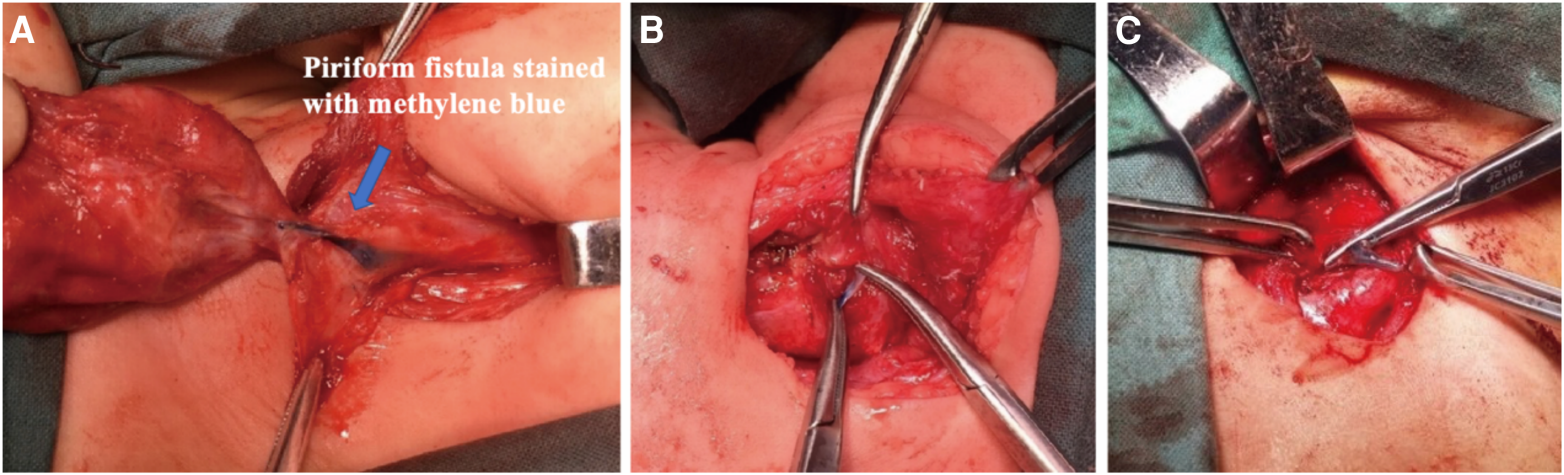
Surgical approaches for pyriform sinus fistulas through hypopharynx. (**A**) A pyriform fistula stained with methylene blue is seen medial to the inferior angle of the thyroid cartilage. (**B,C**) The fistula was easily ligated and excised.

The patients were positioned supine on the table with their necks hyperextended following induction of general anesthesia via endotracheal intubation, and a direct laryngoscope was deployed to locate and confirm the internal orifice (Figure 1). Next, a blunt-tip gastric tube was inserted, and methylene blue was slowly injected into it until the methylene blue overflowed from the internal orifice. The design of skin incisions depended on the cervical scar, using the previous surgical incision whenever possible. In general, the incision was made to include all scar tissue or the external orifice and was elongated bilaterally to allow adequate flap elevation. If there was no incisional scar or external orifice in the neck, a transverse incision was made at the level of the cricothyroid membrane on the affected side. The line alba cervicalis was incised, and the sternocleidomastoid muscle and anterior cervical strap muscles were contoured and retracted laterally. If necessary, the superior pole of the thyroid lobe was retracted medially, and the carotid artery triangle was adopted as the surgical approach. The inferior angle of the thyroid cartilage was covered by the cricothyroid muscle, and the inferior pharyngeal constrictor muscle was then dissected from the oblique line of the thyroid cartilage. Occasionally, the posterior portion of the thyroid cartilage needs to be removed to obtain better exposure, but it should be left intact at least 1 cm above the inferior angle of the thyroid cartilage to avoid injury to a recurrent laryngeal nerve. At this point, the recurrent laryngeal nerve was identified until it entered the larynx around the cricothyroid joint. The posterior cricoarytenoid muscle was an appreciated landmark to remind the surgeon of the possibility of injury to the recurrent laryngeal nerve. In some patients, the inflamed and fibrotic tissues surrounding the inferior cornu must be removed to expose the lesion. The fistula could be identified easily after the dye injection (Figure 2) and was then pursued upward until it reached its dilated portion, namely, the apex of the pyriform sinus. The fistula was ligated and excised as high as possible, followed by excision of the distal portion of the fistula with or without the fibrous cyst. The thyroid cartilage plate was reduced, the fistula transitional segment lateral to the thyroid cartilage plate was dissected, and the fistula cyst, inflammatory mass, and part of scar tissue caused by infection were completely removed. There is no need to perform extensive partial thyroidectomy, the subcutaneous tissue and skin of anterior cervical muscle were sutured by layer, and the drainage was performed. Feeding via nasogastric tube was performed on the first day after the operation, and oral feeding was administered on the fifth to seventh postoperative day.

All included patients were treated according to the protocol described in the above methodology. Cure was defined as meeting the following two criteria: (1) absence of postoperative symptoms such as neck swelling, pain, fever, and/or painful swallowing and (2) absence of infectious lesions or residual fistula in the neck, as confirmed by ultrasound examination conducted 6 months after surgery. Recurrence is determined if the abovementioned clinical symptoms occur in the neck during follow-up or if infectious lesions or residual fistula are detected in the neck through ultrasound examination.

## Results

**Figure 3:**
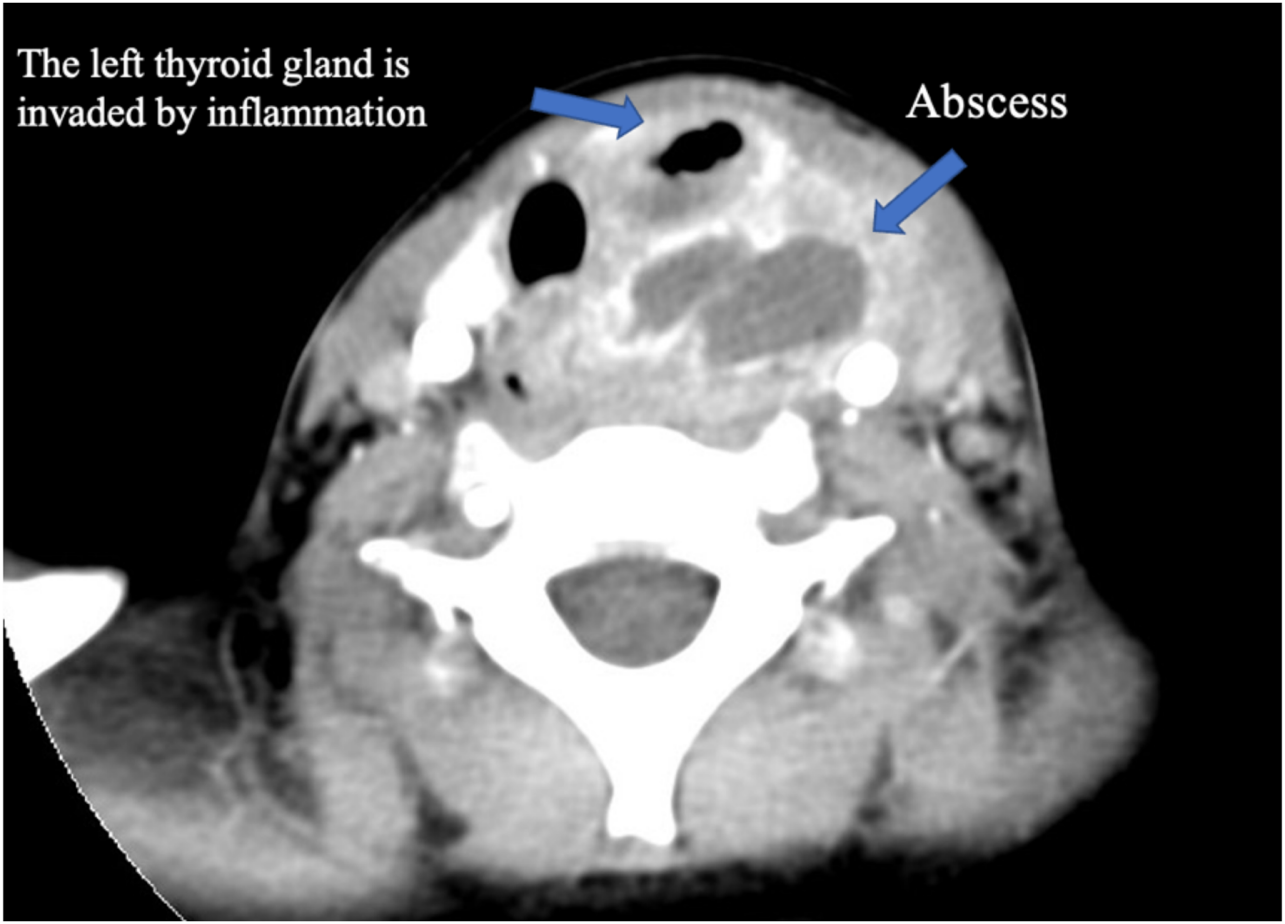
Enhanced CT of the neck. A hypodense, borderline enhancing abscess on the left side of the neck, with invasion of the left thyroid gland and pneumatization.

**Figure 4:**
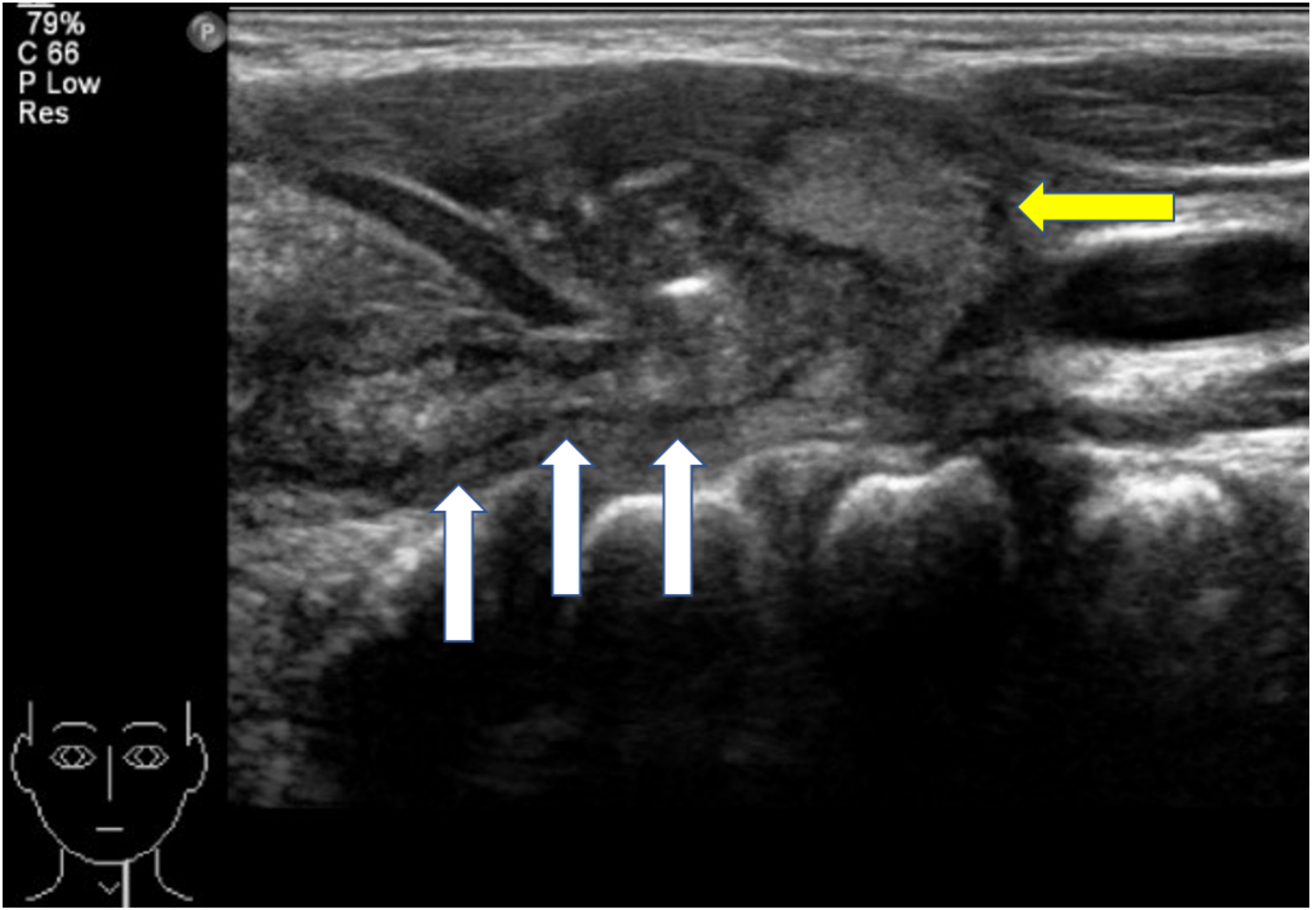
Ultrasound of the neck. The diameter of the fistula is significantly thickened, and the boundary between it and the surrounding tissues is unclear. White arrow, fistula; yellow arrow, left thyroid lobe.

In this study (Table 1), we included 71 patients with PSF, of whom 38 were female and 33 were male. Their ages ranged from 20 days old to 13 years old (average age, 5.4 years old; median age, 6 years old). Clinical presentations included neck abscess (*n* = 59, 83.10%), acute suppurative thyroiditis/thyroid abscess (*n* = 6, 8.45%), and neck mass (*n* = 6, 8.45%). Failed open surgical excision was performed in nine patients at other hospitals. Incisional drainage was performed in 37 patients who had signs of skin redness and tenderness around the mass, along with abnormal WBC counts and CRP levels. Six cases were misdiagnosed as acute suppurative thyroiditis, three cases were misdiagnosed as thyroglossal duct cysts, one case was misdiagnosed as lymphangioma, and two cases were misdiagnosed as dermoid cysts. All 71 patients had fistula found on the left side, while 2 patients had fistula found on both sides. Routine preoperative examinations included an electronic laryngoscope, barium esophagography, ultrasound, and cervical intravenous contrast-enhanced CT or MRI to further evaluate the abnormality, its cervical extent, and its relationship to vital structures. Contrast-enhanced CT and ultrasound examination were performed on all patients. Cervical contrast-enhanced CT revealed soft tissue cellulitis, abscesses, and large cystic lesions containing air and fluid. In 58 patients (81.69%), the neck infections resulted in abscess formation, involving one side of the thyroid gland. The thyroid gland on the affected side is ambiguous and uneven in density, suggesting inflammatory thyroid disease (Figure 3). Ultrasound revealed an inflammatory mass in the neck. Focal hypoechoic lesions were found within the inflammatory mass, which was consistent with local abscess formation. During the ultrasound study, 47 patients (47/71, 66.20%) were diagnosed with PSF directly with direct tubular structures connecting from the hypopharynx to the neck subcutaneous (Figure 4). MRI and barium esophagography also had a satisfactory positive predictive value (5/7, 71.43%, and 3/4, 75%, respectively) but were not widely used in this series. Fine-needle aspiration (*n* = 5) did not contribute to the diagnosis in any of the cases. Electronic laryngoscopy revealed the internal orifice of fistulas in the left pyriform sinus in only six patients (8.45%), while the internal orifice was successfully found in all patients using an intraoperative direct laryngoscope. The final diagnosis was made by direct laryngoscopy of the internal orifice under general anesthesia.

**Table 1:** Summary of data from the 71 patients with pyriform sinus fistula.

Characteristic	Number	%
Sex		
Male	33	46.48%
Female	38	53.52%
Side of anomaly		
Left	69	97.18%
Right	0	0.00%
Bilateral	2	2.82%
Initial presentation		
Neck abscess	59	83.10%
Neck mass	6	8.45%
Acute suppurative thyroiditis	6	8.45%
Imaging		
Barium esophagography	3/4	75%
Computed tomography	58/71	81.69%
MRI	5/7	71.43%
Sonography	47/71	66.20%
Outcome		
Recurrence	0	0.00%
Temporary vocal fold motion impairment	2	2.82%
Esophageal perforation	2	2.82%

Esophageal perforation occurred in two patients during the dissection of PSF, and the esophagus was repaired in time during the operation. After surgery, the two patients were fed via nasogastric tube for 2 weeks and recovered well after the operation. Two patients developed temporary hoarseness (2/71, 2.82%), which resolved 3 months later. All the other patients had no postoperative complications, such as dysphagia, parapharyngeal abscess formation, and incision infection. During a median follow-up of 37 months (13–60 months), there was no recurrence of neck mass was detected after hospital discharge. Ultrasound was performed during the follow-up period, where no signs of fistula recurrence occurred in any of the operative patients.

## Discussion

**Figure 5:**
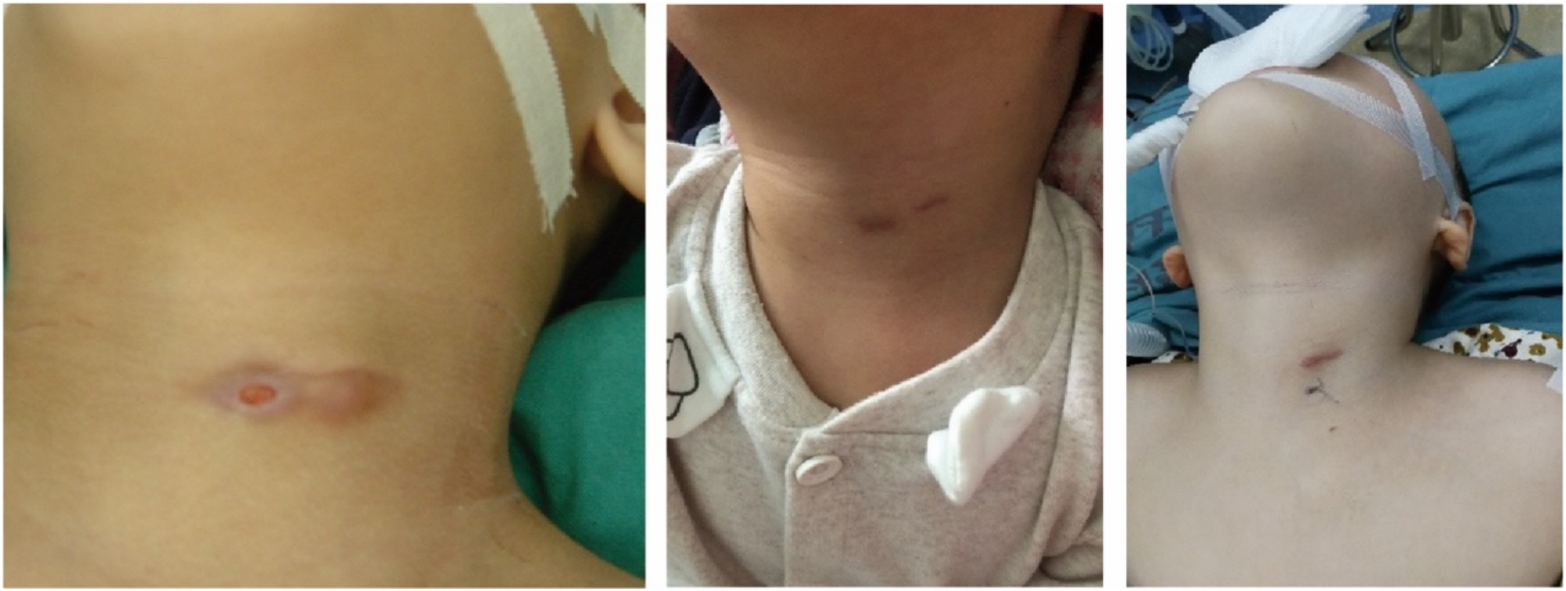
External fistulas caused by surgical incision and drainage.

PSF is characterized by incomplete obliteration of the third or fourth pharyngeal pouch connected to the pyriform sinus, and the clinical presentations of PSF vary with age (5–7). Neonates may present with a non-infectious cervical cyst, which can compress the trachea and cause stridor and respiratory distress. In contrast, PSF presents in young children as recurrent neck abscesses or acute suppurative thyroiditis following an episode of upper respiratory infection. In recent years, the incidence of PSF in children has increased significantly (8), which is partly related to increased awareness of PSF among clinicians. As is known to all, the thyroid is well resistant to infections due to its anatomic and physiologic characteristics, and diagnosis of PSF should be highly suspected in children with acute suppurative thyroiditis, especially on the left neck. Both barium esophagography and laryngoscopy were considered to be the most effective modalities for the diagnosis of PSF (9). PSF can be confirmed by barium esophagography with long and thin tubes extending downward from the tip of the pyriform sinus on the affected side or by electronic laryngoscopy examination with fistula in the pyriform sinus, which is the most common examination method used today. In this study, barium esophagography was not used as a routine examination, due to the obstruction of inflammatory scar tissue in the fistula and swallowing coordination. This method makes it difficult to detect the fistula and is prone to false negatives. Electronic laryngoscopy often presents false negatives as well due to edema of the tissues around the fistula or closure of the fistula during the infection period. In this group, only six patients had PSF internal orifice preoperatively found by electronic laryngoscopy (9.83%). During the operation, the internal orifice was found in the pyriform sinus by direct laryngoscopy in all patients under general anesthesia. This suggests that the internal orifice is not easily visualized due to the mucosal folds or some secretions trapped in the pyriform sinus when the patient is awake. Under general anesthesia, a direct laryngoscope can directly reach the pyriform sinus, open the mucosa, absorb the secretions, and fully expose the pyriform sinus with a good stretching effect on the mucosa folds. According to the authors’ experience, during the process of electronic laryngoscopy in the acute infection stage, slightly pressing the swelling of the neck and spillage of pus or bubbles from the pyriform sinus can increase the positive rate of the examination. In our clinical experience, children with pyriform fistulas have internal fistulas, but almost all of them are born without external fistulas, and all external fistulas are secondary external fistulas caused by surgical incision and drainage of pus after abscess formation (Figure 5). In this case, CT and (MRI) help make a correct diagnosis and facilitate the determination of the course and proximity of the fistula, but both have certain limitations. Therefore, the combination of multiple examination methods, including direct laryngoscopy, can improve the accuracy of diagnosis.

Treatment during the acute inflammatory phase is mainly based on anti-inflammatory and abscess incision and drainage, while surgical excision of the fistula or partial excision of the thyroid gland, endoscopic electrocautery, chemical cautery, CO_2_ laser, and low-temperature plasma radiofrequency ablation are feasible during the non-inflammatory period (10–12). However, all have the problem of recurrence after surgery. According to a large systematic review of data by Derkes et al. (8), the postoperative recurrence rate was as high as 14% for resection of the fistula alone and 18% for cautery only. Josephson et al. (13) reported 147 patients with pyriform fistula treated endoscopically, of whom 25% showed recurrence after surgery. According to the authors’ experience, endoscopic low-temperature plasma radiofrequency ablation for treating pyriform fistula has the advantages of being minimally invasive and easy to perform, and endoscopic treatment of pyriform fistula can be used preferentially for primary patients with inconspicuous skin scarring of the neck or intact skin (14). However, for patients with a large mass in the neck causing compression symptoms, with repeated incision and drainage of pus with more obvious scars and external fistula openings in the neck, with or recurrence cases following repeated cauterization, alternative approaches may be necessary. Therefore, endoscopic surgery has its limitations for these types of patients. Given the various disadvantages of the currently used procedures, it is necessary to improve the surgical approach. To address the above situation, we performed a modified direct laryngoscopic-assisted radical treatment of the pyriform fistula via hypopharyngeal, which allows for complete excision of the fistula and simultaneous management of the inflammatory mass formed by the fistula infection and the keloid formed by repeated incisions to drain the pus, preventing recurrence.

Clinically, PSF mostly originates at the tip of the pyriform fossa, penetrates the cricothyroid muscle, encounters subpharyngeal constriction, and penetrates obliquely medially or laterally in the cricothyroid joint. It is often confined around the thyroid gland or extends down through the thyroid gland to the root of the neck (15). Additionally, the fistula travels in close relationship with the external branches of the superior laryngeal nerve, recurrent laryngeal nerve, thyroid gland, and carotid sheath. Due to its complex anatomy, complete excision of the fistula is not only difficult but also has many complications (5, 9), such as vocal cord paralysis (3.71%–33%), pharyngeal leak, salivary leak, wound infection, facial nerve paralysis, cricothyroid dislocation, cervical scar deformity, posterior group cranial nerve paralysis, and Horner's syndrome. Some scholars have suggested that for patients with multiple incisions and drainage or surgery, extensive scar formation in the neck, or closed fistulas with unrecognizable adhesions to granulation scar tissue may require function-preserving elective neck debridement (16, 17). Due to recurrent inflammatory infections, complex neck anatomy, and inappropriate surgical management, recurrence rates after conventional open surgery are as high as 4.9%–50% (8, 18, 19), and complications are even more common in children of younger ages (16, 18). It is evident that the knowledge of the fine anatomy of the cricothyroid region determines whether the fistula can be removed completely and reduces the incidence of complications. In the authors’ experience, the fistula is more easily identified after staining by injecting methylene blue into the internal fistula opening with the assistance of direct laryngoscopy, and the fistula is then identified in all our surgical cases by injecting methylene blue. Reports have described the use of methylene blue to trace fistulas intraoperatively, but due to repeated infection of the fistula, the fistula is slender and tortuous, and the injection of methylene blue through the internal fistula opening may not reach the root of the fistula. If the root of the fistula is blindly searched intraoperatively, it is easy to damage the superior pole of the thyroid blood vessels and the laryngeal recurrent nerve that penetrates the pharyngeal constriction muscle, leading to complications such as postoperative hoarseness and dysphagia. There are reports that the fistula was inadvertently broken during the injection of methylene blue tracer and methylene blue accidentally leaked into the operative field, making it difficult to find the fistula intraoperatively (20), so we suggest that methylene blue should not be injected roughly into the fistula through the gastric tube and that a small amount of methylene blue should be injected into the fistula in a pulsatile manner. Xiao et al. (21) classified PSF into three types based on the relationship between the fistula alignment and the inferior angle of the thyroid cartilage: Type I, fistula to the anterior thyroid cartilage (28.5%); Type II, fistula to the inferior thyroid cartilage (23.4%); and Type III, fistula to the posterior thyroid cartilage (48.1%). We found this anatomical feature provides the basis for the design of the surgical approach, i.e., the fixed landmark, the inferior angle of the thyroid cartilage, is the anatomical landmark for finding the root of the fistula. An adequate understanding of the embryological and anatomical basis of PSF and clarification of the relationship between the fistula and the inferior angle of the thyroid cartilage will facilitate complete intraoperative excision of the fistula. Because the PSF fistula root is closely related to the inferior angle of the thyroid cartilage, the fistula root is fixed here through the inferior pharyngeal constriction muscle, and the surgical pathway with the inferior angle of the thyroid cartilage as the bony landmark is easy to locate the fistula and shorten the surgical pathway. In addition, the inferior horn of the thyroid cartilage is a cartilaginous component, which is different from the surrounding muscle tissue, and the intraoperative bony anatomical landmarks make it easier to expose the inferior horn of the thyroid cartilage and the root of the pyriform fossa next to or below it. Additionally, the root of the PSF fistula is thicker, and the fistula has been internally stained with methylene blue stain before surgery, making it easier to distinguish and find the fistula intraoperatively.

In contrast to the conventional external cervical fistula removal via the external fistula opening, this procedure allows the ligation of the fistula via the hypopharynx through the inferior angle of the thyroid cartilage, which can further treat the fistula in the medial thyroid cartilage larynx based on the lateral fistula of the thyroid cartilage plate and achieve the purpose of high ligation, thus completely removing the fistula of the pyriform fossa and preventing the formation of a stump leading to recurrence. The postoperative follow-up requires observation of the patient's neck wound for signs of infection, such as redness, swelling, and pain, and the presence of inflammatory masses or residual fistulas can be observed by ultrasound of the neck during the follow-up period, which is recommended to be more than 1 year. There is no recurrence in this series. Only two patients had transient hoarseness after surgery, which resolved after 3 months, and the rest of the patients had no postoperative complications such as dysphagia, parapharyngeal abscess, or incisional infection.

The limitations of this study include its retrospective nature, small sample size, and short clinical follow-up. Hence, large, multicenter prospective and controlled clinical studies with long-term follow-up are necessary.

## Conclusion

This novel modified surgical approach, which is based on anatomical principles, discards the traditional method of removing external fistulas by introducing an external cervical route via the hypopharyngeal route, which allows easy localization of the incision and easy exposure of the fistula. For patients with repeated incision and drainage, scar deformity or keloid formation in the neck, large cysts, inflammatory occupying lesions in the neck, or obvious external fistulas affecting the appearance of the neck, this approach allows for the removal of both surgical scars and occupying lesions, which is more advantageous than endoscopic treatment, and can be the final treatment option for patients undergoing recurrent endoscopic treatment.

## Data Availability

The original contributions presented in the study are included in the article/Supplementary Material, further inquiries can be directed to the corresponding author/s.
